# Causes and Consequences of Past and Projected Scandinavian Summer Temperatures, 500–2100 AD

**DOI:** 10.1371/journal.pone.0025133

**Published:** 2011-09-22

**Authors:** Ulf Büntgen, Christoph C. Raible, David Frank, Samuli Helama, Laura Cunningham, Dominik Hofer, Daniel Nievergelt, Anne Verstege, Mauri Timonen, Nils Chr. Stenseth, Jan Esper

**Affiliations:** 1 Swiss Federal Research Institute WSL, Birmensdorf, Switzerland; 2 Oeschger Centre for Climate Change Research, Bern, Switzerland; 3 Climate and Environmental Physics, Physics Institute University of Bern, Bern, Switzerland; 4 Arctic Centre, University of Lapland, Rovaniemi, Finland; 5 School of Geography and Geosciences, University of St Andrews, St Andrews, Scotland; 6 The Finnish Forest Research Institute, Rovaniemi, Finland; 7 Centre for Ecological and Evolutionary Synthesis CEES, University of Oslo, Blindern, Norway; 8 Department of Geography, Johannes Gutenberg University of Mainz, Mainz, Germany; Humboldt University, Germany

## Abstract

Tree rings dominate millennium-long temperature reconstructions and many records originate from Scandinavia, an area for which the relative roles of external forcing and internal variation on climatic changes are, however, not yet fully understood. Here we compile 1,179 series of maximum latewood density measurements from 25 conifer sites in northern Scandinavia, establish a suite of 36 subset chronologies, and analyse their climate signal. A new reconstruction for the 1483–2006 period correlates at 0.80 with June–August temperatures back to 1860. Summer cooling during the early 17th century and peak warming in the 1930s translate into a decadal amplitude of 2.9°C, which agrees with existing Scandinavian tree-ring proxies. Climate model simulations reveal similar amounts of mid to low frequency variability, suggesting that internal ocean-atmosphere feedbacks likely influenced Scandinavian temperatures more than external forcing. Projected 21st century warming under the SRES A2 scenario would, however, exceed the reconstructed temperature envelope of the past 1,500 years.

## Introduction

Annually resolved, large-scale temperature reconstructions of the last millennium rely on a handful of predictor records, which often reflect heterogeneous patterns of local- to regional-scale climate variability [1]. Due to the small number of highly resolved proxy data available prior to the Little Ice Age (LIA; ∼1350–1850 AD), the few tree-ring width (TRW; mm/year) and maximum latewood density (MXD; g/cm^3^) chronologies from northern North America and northern Eurasia that span the last millennium, become increasingly important further back in time. Long-term extra-tropical temperature estimates typically incorporate the same proxy records and use similar reconstruction methodologies [2]. For instance, millennium-long tree-ring composite data from the Torneträsk region in northern Sweden (e.g. [3] and references therein) contribute to most large-scale temperature reconstructions [4], thus clearly impact our understanding of the spatial extent, absolute timing and relative amplitude of the Medieval Climate Anomaly (MCA; ∼900–1300 AD). At the same time, it appears particularly important to note that multi-decadal variations in surface air and sea surface temperature (SAT and SST) across the North Atlantic/Scandinavian sector are dominated by internal climate variability [5]–[9], which may does not always agree with short-term events and long-term trends observed in other areas or more generally larger spatial scales. Consequently, reconstructions of hemispheric to global average temperatures that partly rely on Scandinavian proxy data, likely contain climatic fingerprints of internal variability that might not be overly representative outside a limited region (e.g. [10]).

One source of internal climate variability across Scandinavia is the Atlantic Multidecadal Oscillation (AMO), which had a periodicity of ∼65–70 years during the last 150 years [11]. Observational and model evidence indicates that the Atlantic Meridional Overturning Circulation (AMOC) plays an important role in driving the AMO [12], which in turn controls summer climate across the high northern latitudes (e.g. [7]). In addition to the AMO, atmospheric circulation modes, such as the North Atlantic Oscillation (NAO), the Arctic Oscillation (AO) or the East Atlantic Pattern (EAP) may influence Scandinavian climate variability on different timescales (e.g. [13]–[15]). Quantifying and distinguishing between such internally driven ocean-atmosphere-land dynamics and externally forced climate variations represents a major challenge for the detection and attribution of anthropogenic-induced climate change (e.g. [6], [8], [16]). The combined assessment of proxy-based climate reconstructions and model-based climate simulations therefore becomes essential for allowing studies to separate externally forced trends from internally driven variations, whenever the climatic signal is distinguishable from noise (e.g. [17]).

This study presents the largest update of regional MXD measurements undertaken to date, allowing modern growth-climate responses to be analysed into the 21st century. We aim to develop a new reconstruction of northern Scandinavian summer temperature variability over the past five centuries, compare this record with existing tree-ring evidence and model output, and disentangle the role of external climate controls from internal ocean-atmosphere oscillations.

## Results

**Figure 1:**
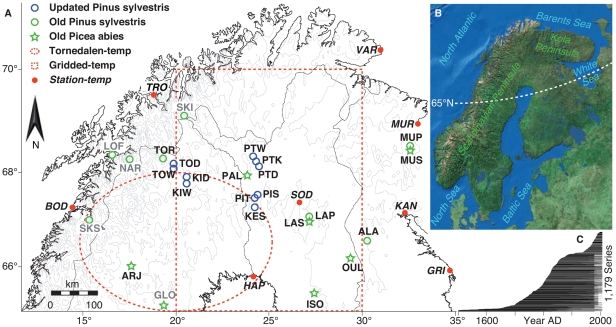
Spatial characteristics of the northern Scandinavian tree-ring network. (**A**) The 25 MXD site chronologies and the eight instrumental stations incorporated within this study. Grey labels refer to five MXD sites from the westerly portion of the network that showed a modest sensitivity to temperature and thus were not included in the final reconstruction. (**B**) Overview of the North Atlantic/Scandinavian sector. (**C**) Temporal distribution of the 1,179 MXD samples during the past five centuries.

The 25 site chronologies show positive correlations with growing season temperatures between April and August (see supporting information (SI) Text S1 for details). The highest correlation is found with June–August (JJA) temperatures, corresponding to intense cell formation (lumen enlargement) and wood lignification (wall thickening). Significant (*p*<0.001) positive correlations with JJA temperatures (0.31–0.83) were obtained from the 36 MXD records including 25 site and 11 subset chronologies (Figure 1–3). Small differences in mean MXD (0.59–0.85 g/cm^3^), but major differences in sample replication (18–1179 series), as well as mean tree age (39–324 years) are found (Figure S1). Less replicated site chronologies generally contain a lower temperature signal. Five site chronologies from the network's western margin, which are based on 22–29 samples only, show modest correlations (Table S1 and Figure S2), and thus were omitted as predictors in the final reconstruction.

**Figure 2 pone-0025133-g002:**
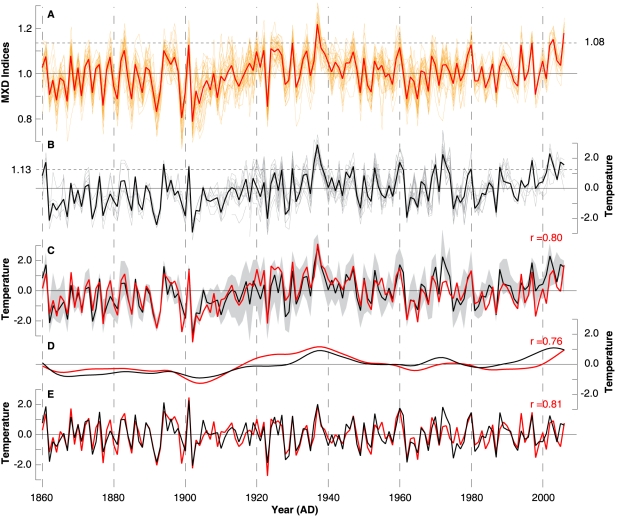
Agreement between high to low frequency variability of the tree-ring proxy and instrumental target data over the past 150 years. (**A**) Site and subsets RCS chronologies (orange) and their mean (red). (**B**) JJA temperatures of eight instrumental stations (grey), and the grid-box mean (black). Dashed horizontal lines and corresponding values indicate the mean of the last decade (1997–2006). (**C**) Instrumental gridded (black) and reconstructed proxy (red) temperatures superimposed on the min/max range of the station measurements (grey shading), expressed as anomalies from 1961–1990, and split into (**D**) 20-year low-pass and (**E**) 20-year high-pass components. Correlation (1860–2006) of the unfiltered and filtered timeseries is shown in red.

**Figure 3:**
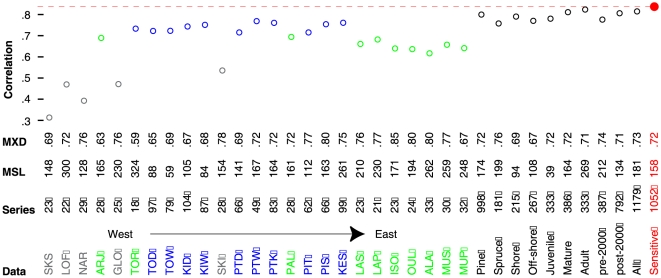
Climate response of the tree-ring network revels significant positive correlations between MXD chronologies and June–August temperatures. Correlation of the 36 MXD site and subset chronologies against Scandinavian JJA temperatures (1860–1977). Information on maximum latewood density (MXD; g/cm^3^), mean segment length (MSL; years), and replication (series) is provided for each dataset. Grey [green/blue] site codes refer to less-sensitive (pre/post-2000) chronologies. The red dot refers to the sensitive subset herein used for reconstruction purpose, with the dashed line highlighting the corresponding maximum correlation with JJA temperature (0.83). Note that all correlations exceed the 99.9% significance level.

Subset replication ranges from 181–1179 series, and is substantially larger than individual site replication (18–104 series). Some differences in the growth-climate response were expected within several subsets that combined physiological (species and age), ecological (shore/offshore), and methodological (sampling design) extremes. Nonetheless, all subset chronologies were strongly correlated with regional summer temperatures (>0.76), suggesting a considerably high degree of common variance within the MXD network. Lower, but still significant (*p*<0.001) correlations of 0.29–0.63 were found between the 36 MXD chronologies and JJA coastal Norwegian SSTs averaged over the 65–70°N and 5–15°E region.

The 36 MXD chronologies reflect similar growth trends over the past ∼150 years (Figure 2A) and show strong cross-correlation with each other (*r*  = 0.72 for 1860–1977). All records contain below average MXD values in the 1860s, ∼1900s, and in the 1960s. Above average values are evident from ∼1920–1950 and during the last ∼15 years. The highest MXD values occur in 1937. The lowest MXD values are observed in 1902. A similar picture derives from the eight instrumental records of JJA temperatures and the corresponding CRUTEM3v grid-box mean (Figure 2B). Cross-correlation between the eight station measurements is 0.70 (1860–1977), which is even below the analogous value of the 36 MXD chronologies. Minimum and maximum JJA values of the individual station records represent a realistic range of local temperatures that can be utilized as a target envelope for the MXD chronologies (Figure 2C). This envelope remains relatively narrow throughout the past 150 years; however, increased spread between the station measurements is somewhat evident during the first three decades of the 20th century. This period coincides with decreased correlations among the individual MXD chronologies. The good fit observed between the proxy and target data also demonstrates that both high and low frequency coherence has been maintained (Figure 2C). The composite record tracks the recent and particularly the earlier warmth of the 1930s, remains within the target envelope, encompasses 70% of northern Scandinavian JJA temperature variations (CRUTEM3v; 1860–2006), and further reflects 36% of coastal Norwegian SST variability (SSTV2; 1860–2002). The correlation between instrumental SAT and Norwegian SST is 0.63 when calculated over the common period 1860–2002, suggesting that SST similarly influences Scandinavian MXD formation and SAT variation. Detailed calibration/verification statistics are summarized in Table 1.

**Table 1:** Calibration and verification statistics indicate proxy/target agreement on all frequency domains.

	Full Calibration	Late Calibration	Early Calibration	Extra Calibration
	1860–2006	1934–2006	1860–1932	1816–1859
**r**	0.80	0.76	0.83	0.89
**(low/high)**	(0.76/0.81)	(0.70/0.78)	(0.89/0.84)	(0.96/0.88)
**DW**	1.55	2.10	1.42	1.73
	Extra Verification	Early Verification	Late Verification	Full Verification
**RE**	0.64	0.64	0.63	0.45
**CE**	0.61	0.39	0.38	0.41

Correlation coefficients of 12 proxy/target pairings, independently computed over different split periods and frequency domains, and for unfiltered and 20-year low- and high-pass filtered data range from 0.70–0.96 with a mean of 0.83 (*p*<0.001). Corresponding Durbin and Watson (DW) [48] statistics range from 1.42–2.10 (Table S3), and RE/CE statistics range from 0.45–0.64/0.38–0.61.

The proxy and instrumental data correlate at 0.80 over 1860–2006. Correlation coefficients remain approximately constant after 20-year high- and low-pass filtering, which is suggestive of near equal fidelity among inter-annual to multi-decadal variability. Nevertheless, a detailed examination of the proxy/target relationship over different frequency domains indicates some decadal-scale disagreements during the early and late 20th century (Figure 2D–E), as well as some discrepancy in the timing of positive extremes. The 20-year low-pass filtered timeseries exhibit slightly higher reconstructed values, relative to measured temperatures, from ∼1915–1945 and slightly lower temperatures from ∼1985–2006. The level, direction and timing of disagreement between the proxy and target records, however, do not indicate any systematic ‘Divergence’ during the 20th century (e.g. [18]).

The proxy reconstruction is found to be less sensitive to warm than to cold extremes (Table S2), a trait shared with other MXD-based temperature reconstructions, which typically better capture cold temperature events (e.g. [3], [19]–[21]). Five of the ten coldest reconstructed and observed summers are common to both records, whereas only two out of the ten warmest years are indicated by both the instrumental and tree-ring data (Table S2). However, it should be noted that both the warmest (1937) and coldest (1902) instrumentally recorded summers are robustly retained by the MXD data.

**Figure 4:**
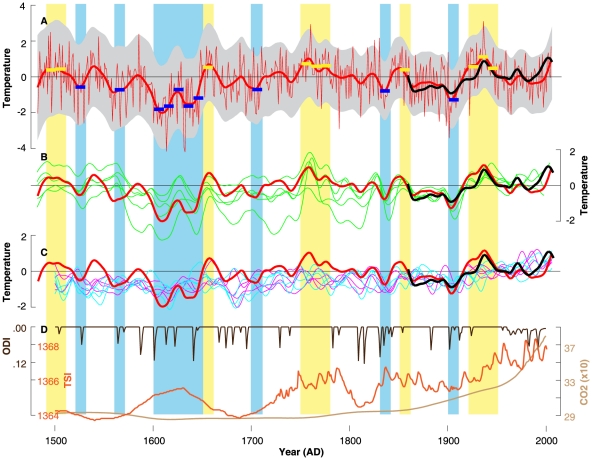
Reconstructed and modelled northern Scandinavian summer temperature variations over the past five centuries. (**A**) Actual (black) and reconstructed (red) JJA temperature anomalies (°C) with error estimates (grey) and the ten warmest and coldest decades superimposed (colour boxes). (**B**) Comparison of the actual and reconstructed temperatures with five existing (green) reconstructions (see Table S3 for details). (**C**) Comparison of the actual and reconstructed temperatures with CCSM3 SAT (pink) and SST (blue) model simulations. Mean and variance of the data are scaled against JJA temperature (1860–2006), expressed as anomalies (1961–1990) and 20-year low-pass filtered. (**D**) Solar (TSI; Total Solar Irradiance), volcanic (ODI; Optical Depth change in the visible band), and CO_2_ (ppm) forcing as used in the model simulation.

The new summer temperature reconstruction spans the period 1483–2006 and reveals inter-annual to multi-decadal fluctuations without clear indication for cooler LIA and warmer recent conditions (Figure 4A), which contrasts evidence from other regions (e.g. [19], [22]) and larger scales (e.g. [1], [23]). Warming is most pronounced around 1500, 1660, 1780, and 1930. Cooling is most distinct during the first half of the 17th century. The ten warmest summers occur sporadically throughout the reconstruction (Table S2). The warmest reconstructed JJA temperature occurred in 1937 (3.11°C), while the coldest reconstructed summer occurred in 1633 (−4.19°C).

Spatial significance of the new reconstruction is approximately restricted to the Scandinavian Peninsula and the Baltic Sea >55°N and <45°E (Figure S3A). This pattern is confirmed by non-significant (*p*>0.1) correlations between the new Scandinavian reconstruction and independent MXD-based summer temperature reconstructions from the Polar Urals, Pyrenees and Alps that range between 0.1 and −0.07 (1483–1989). Spatial correlations with SST are positive for the surrounding northeast Atlantic, as well as the North and Baltic Seas. Positive correlations with the western part of the North Atlantic around 60–70°W and 30–40°N are also observed (Figure S3B), however, probably reflect a dynamic seasonal structure of surface air pressure rather than a continual pattern of water circulation. This assumption is supported by spatial field correlations between our new reconstruction and gridded (5°×5°) June–August SLP (HadSLP2r; 1860–2006) (Figure S4).

Below-average pressure anomalies centred over the Baltic Sea are associated with the 20 coldest reconstructed summers that exhibited in northern Scandinavia between 1659 and 1999 (Figure S5A). These negative pressure anomalies are teleconnected with positive pressure anomalies over the Bay of Biscay, thus describing a European north/south dipole structure related to anomalous cold westerly winds directed towards Scandinavia. Anomalously high northern Scandinavian summer temperatures, on the other hand, match a well-defined pattern of positive pressure anomalies over the Baltic Sea, and negative anomalies west of Iceland (Figure S5B). The southern European counterpart of negative pressure extends over the Mediterranean basin.

Our northern Scandinavian JJA temperature history agrees well with a TRW-based reconstruction of summer AO indices [24] (*r* = 0.39 between 1650–1975; Figure S5C). Amplification of the strong positive agreement, however, may possibly result from overlapping data within this study and the AO estimate, and the fact that the AO study considered overall temperature sensitive proxy data [24]. Correlations between the temperature and AO reconstructions increase to 0.61, 0.70 and 0.73 after 10-year, 20-year and 30-year low-pass filtering, respectively. This frequency-dependence supports the strong multi-decadal coherency between variations in northern European summer temperature and high latitudinal pressure fields.

Comparison between our new record and five existing northern Scandinavian summer temperature reconstructions reveals a sound picture over the past two centuries (Figure 4B), but describes some offset prior to ∼1800. It should be noted that these records are not methodologically independent and include some common data (Table S3). The five existing records show slightly lower correlations (*r* = 0.52–0.80) with gridded JJA temperatures (CRUTEM3v; averaged over 65–70°N and 20–30°E; 1860–1970) than the new reconstruction (*r* = 0.85). The five existing records express an average cross-correlation of 0.59 for the 802–1970 common period. Our new reconstruction correlates with the individual existing reconstructions between 0.42 and 0.76 (1483–1970), and at 0.74 with their average. After 20-year low-pass filtering, this value slightly decreases to 0.67. The five individual records fall within the uncertainty range of our new reconstruction and portray a similar sequence of warm and cold periods.

Comparison of our reconstruction with the SAT and SST of four ensemble simulations reveals that the model output falls within the proxy uncertainty but contains marginally less amplitude (Figure 4C). Some offset between cooler reconstructed and warmer simulated temperatures is found during the last half of the 20th century with the proxy values better matching the instrumental target. Comparison of the reconstructed and simulated temperature variations and trends with external solar, volcanic and CO_2_ forcing (as used in the model) retains a rather weak link over the past 500 years (Figure 4D). Since systematic perturbations of external forcing are not evident in the reconstructed and simulated Scandinavian temperature history, internal climate dynamics must have played an important role in determining past summer temperatures within this region. It should be noted that the four SAT (SST) model ensembles correlate on average at 0.50 (0.48) with each other (1500–2100). Significant differences in coherency are found before and after 1850 for the SAT (0.04 and 0.56) and SST (0.09 and 0.51) ensemble members. A similar pattern with overall higher correlations is obtained after 20-year low-pass filtering. Early/late correlation averages are 0.21/0.92 for SAT and 0.17/0.90 for SST, indicating less externally forced agreement prior to the industrial era, for instance. Correlations between our new reconstruction and the four SAT (SST) ensemble members (after 20-year low-pass filtering) average at 0.37 (0.24) over the full 1483–2006 period of overlap, with only small differences before and after 1850.

**Figure 5:**
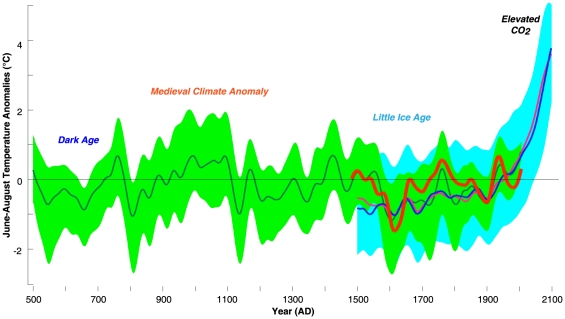
Proxy and model evidence of northern Scandinavian summer temperature variability from 1500–2100 AD. Reconstructed (red this study and green mean of five existing records) and projected (pink surface air and blue sea surface) temperature anomalies of the 1961–1990 reference period and 60-year low-pass filtered. Green and blue shadings indicate the uncertainty envelopes derived from the annual minimum and maximum values of the various proxy and model data, respectively.

The combined proxy/model approach allows variations in pre- and post-industrial northern Scandinavian summer temperature to be assessed over the period 500–2100 (Figure 5). The tree-ring data indicate lower temperatures before ∼700 and around ∼800, and then again in the early 17th century commonly known to be part of the LIA. The first warm spell is recorded at ∼760, a prolonged interval of generally warm summers occurred between ∼1000 and 1100, warm summers also appeared ∼1430, ∼1770, and again in the 1930s and the 1970s. The early values, however, should be interpreted cautiously, because the amount of available TRW and MXD measurement series used in five northern Scandinavian proxy records decreases back in time. Nevertheless, our synthesis clearly demonstrates that summer temperatures have previously been as markedly warm as the observed 20th century conditions including the high temperatures of the 1930s and 1970s. Despite this, the model output further suggests that, under the SRES A2 scenario, temperatures will exceed the range of past variability from ∼2030 onwards (Figure 5).

## Discussion

Our MXD compilation represents a major update in one of the key regions in a circumpolar network [20], [22], enabling the relationship between forest productivity and recent climate variability to be evaluated into the 21st century. The ability of MXD chronologies from northern Scandinavian conifers to capture the timing, amplitude and geographical distribution of recent temperature fluctuations is evidently confirmed. There is no indication for any systematic ‘Divergence Issue’ (e.g. [18]), namely the failure of tree growth to track high and low frequency temperature changes. Furthermore, our results reliably reproduced not only inter-annual temperature variations, but also long-term trends over a well-defined spatial domain (e.g. [20], [22]). Such findings are consistent with updated tree-ring compilations from northern Siberia [25] and the European Alps [26], although the actual reconstructions themselves reflect different signals of regional climate fluctuations. Constraints in capturing low frequency temperature variability that could potentially result from either the tree-ring data used or the detrending methodology applied, have been addressed by the inclusion of young trees resulting in an even distribution of series start dates throughout time and by the performance of RCS detrending (for details see below). Uncertainty ranges associated with the resulting temperature reconstruction have been estimated via cumulative error estimates (see below), which likely resulted in a rather conservative uncertainty range. The effects of site location, shore position, species-specific response, tree age, sampling design, growth standardization, and proxy calibration have been considered, and are expressed by the temporal variation of error estimates.

A slight offset between cooler proxy reconstructions and warmer model simulations is observed during the latter half of the 20th century (Figure 4). This discrepancy is potentially due to slightly overestimated temperatures within the ensemble members, as anthropogenic sulphate aerosols have not been included in the model forcing. A sensitivity experiment, including this forcing, though resulted in less warming during the 20th century [15]. It is further noted that both reconstructed and simulated Scandinavian SAT show only weak responses to natural external forcing, which obviously indicates the importance of internal climate dynamics within this region. As shown by [15], variations in SST, which in turn are connected to the North Atlantic Thermohaline Circulation, must be considered as important drivers of multi-decadal climate variability across the North Atlantic/European sector and particularly over the Scandinavian study region. These internal atmosphere-ocean variations might also be strong enough to largely mask solar, volcanic, and even anthropogenic signals as suggested by the herein reconstructed and simulated temperatures of the past 500 years. Our results highlight the strength of a combined proxy/model approach to examining climatic variability, which extends the analytical skill of either using proxy reconstructions or model simulations only. The joint consideration of backward reconstructing and forward modelling further offers longer timescales to be evaluated. The Scandinavian records, however, differ from the situation commonly observed in Central Europe where impacts of solar and volcanic forcing on past temperature change have been undoubtedly identified (e.g. [19], [27]). Based upon the available findings, it appears likely that the ocean may play a role in generating low frequency temperature variability.

Another important source of internal low frequency variability are atmosphere dynamics that comprise dominant pressure patterns, such as the AO, the NAO and the EAP, which in turn are often coupled with the underlying climate system (e.g. [5], [28]). These pressure patterns may possibly exhibit linkages to temperature and precipitation fluctuations at decadal to multi-decadal timescales north of ∼20°N. This suggestion is supported by the agreement between our new Scandinavian temperature history and a previous reconstruction of the summer AO [24].

Nevertheless, it is premature to conclude that globally observed effects of recent anthropogenic warming on ecology [29] may be less intense over Scandinavia, where internal climate variability likely moderated and modulated some of the latest temperature increase observed in other regions and at larger scales. Despite their dominance in large-scale temperature reconstructions, initial tests suggest that such hemispheric approaches are not particularly sensitive to the inclusion or exclusion of Scandinavian tree-ring data especially after ∼1300 AD (Figure S6). Caution is advised as more detailed analyses at the hemispheric-scale are necessary to better differentiate between regional and lager scale climate change and even disentangle the relevant forcing agents at play. If Scandinavian summer temperatures indeed exhibit less post 1980s warming relative to earlier warm periods than many other regions across the Northern Hemisphere, however, still needs to be quantified.

The unprecedented degree of projected anthropogenic warming under the SRES A2 scenario as described by the modelling component of this study, would though imply severe alterations to ecosystem functioning and productivity, as well as trophic amplifications across a broad range of marine and terrestrial habitats and taxa [30], [31]. Long-term stability of boreal population dynamics, sustainable food webs and biodiversity conservations are thus at risk [32], [33]. Temperature proxies derived from oxygen isotopic analyses of foraminifera located along the Norwegian margin offer relatively high-resolution evidence on comparable timescales and thus have been used to link terrestrial and marine ecosystem responses to climate changes [34]. Previous temperature reconstructions have relied on physical-chemical relationships, namely that a 0.26‰ shift in the oxygen isotopes reflects a 1°C change in water temperature [34]. More recent endeavours have demonstrated the potential to develop shell-growth increments of *Artica islandica* in Norwegian coastal waters, which can contain well-defined climate signals [35]–[37]. Currently, these records only span several centuries. Nevertheless, it may be possible to extend them further, potentially even over the entire Holocene. If this would be achieved, the combination of such archives and the new MXD reconstruction presented here would enable further elucidation of the influence of ocean dynamics and internal forcing on temperature variability within this region.

Increased efforts to obtain new high-resolution proxy archives in tandem with an ensemble of model simulations is recommended not only to disentangle effects of external climate forcing from internal climate variability, but also to improve our understanding of associated spatiotemporal shifts in ecosystem responses to climatic variations. The expected outcome of such work would be relevant not only for climatologists, but also for ecologists, epidemiologists, oceanographers and even economists throughout the multiple effects of climate change on biological resources.

## Materials and Methods

No additional sampling permits beside those organized by Mauri Timonen (MT; among the co-authors) were required to develop the herein presented MXD dataset (Dr. Yrjö Norokorpi, Area Manager, Natural Heritage Services of Metsähallitus, granted 15.09.2006 permission for tree-ring sampling to MT and the WSL research team of UB, DF, DN and JE). A total of 1,179 tree-ring core or disc samples were collected between 1978 and 2006 at 25 Scandinavian conifer sites located north of 65°N (Figure 1). Eighteen site chronologies consisted of pine (*Pinus sylvestris*), while seven were based on spruce (*Picea abies*). All of the 1,179 MXD measurement series were processed at the Swiss Federal Research Institute WSL (see [38] for methodological details). Pith-offset – the amount of years between the innermost ring and the expected germination age – was estimated for each core sample to range between 0 and 170 years. Non-climatic, i.e. biological-induced age trends were removed from the raw MXD measurement series using the Regional Curve Standardization (RCS) method on a site-by-site basis [39]. Series were first aligned by cambial age; a mean of the age-aligned series was then calculated and smoothed with a cubic spline of 10% the series length [40]. The resulting timeseries is termed the Regional Curve (RC) and used for detrending, i.e. deviations of the individual measurements from the RC were calculated as ratios. Dimensionless indices were transformed back to calendar years, and averaged per site using a robust bi-weighted mean. The variance of the subsequent mean chronologies was adjusted for changes in sample replication and inter-series correlation [41], with only periods whose replication exceeded 10 series were analysed further.

Gridded CRUTEM3v data (1860–2006) [42] averaged over 65–70°N and 20–30°E were employed as predictor variables for the reconstruction of Scandinavian summer temperatures (SI). Variability resulting from differences in site location, species-specific responses, lakeshore position (i.e. the distance between trees and the nearest lake), tree age, sampling design, standardization methodology, and model calibration was integrated in the estimated reconstruction error (see SI for details).

Eight homogenized station records (also included in the CRUTEM3v data) with continuous temperature measurements spanning >70 years were selected from the Global Historical Climatology Network (GHCN-adjusted; Figure 1 and Table S1), to estimate a likely range of northern Scandinavian temperature variations. Differences between local station records were used to calculate a data target envelope used for comparison with the MXD chronologies. The combined Tornedalen record was considered for extra-verification back to 1816 [43], and gridded SSTs from coastal Norway (65–70°N and 5–15°E, 1892–2002) were utilized for comparison (SSTV2) [44]. Correlations with gridded SAT and SST data, as well as comparison with MXD-based summer temperature reconstructions from the Polar Ural [25], the Pyrenees [45], and the Alps [19], were performed to evaluate the spatial signature of Scandinavian temperature variability. A composite analysis of gridded 500-hPa geopotential height field data [46] was used for the reconstructed 20 coldest and warmest Scandinavian summers of the period 1659–1999. This analysis allowed the assessment of possible impacts of large-scale atmospheric circulation patterns on more regional northern Scandinavian summer temperature extremes. A suite of five published tree ring-based summer temperature records from northern Scandinavian was gathered for comparison with our new record (Table S3). The five chronologies preserve inter-annual to multi-centennial variability and were rescaled against the same instrumental target record (CRUTEM3v) as the newly developed temperature reconstruction presented in this study. Finally, we used an ensemble of four transient simulations from the coupled Community Climate System Model version 3 (CCSM3) [47], covering the 1500–2100 period to derive proxy-independent temperature variations and run proxy/model comparisons (see SI for details).

## Supporting Information

Text S1 Supporting information.
(DOC)

Figure S1 Temporal coverage of the 1,179 MXD series, of which 387 were developed before 2000 AD with 792 added in 2006, covers the entire Scandinavian peninsula north of 65°N and has a mean segment length (MSL) of 161 years with an maximum latewood density (MXD) of 0.72 g/cm3 (inset denotes the relationship between tree age and MXD value). Site chronologies start between 1400 and 1844, and their autocorrelation ranges from 0.23–0.50.
(EPS)

Figure S2 Correlation of the 25 MXD site chronologies after RCS detrending (circles) with monthly temperature means (CRUTEM3v) computed over the common 100 yr interval (1860–1977). Results are classified into 20 sensitive (green) and 5 less sensitive (grey; GLO, LOF, NAR, SKI, SKS) sites, with the horizontal lines referring to their average response.
(EPS)

Figure S3 (**A**) Correlation (r>0.3) of the reconstruction against gridded JJA SAT (1901–2006) and four MXD-based summer temperature records: 1 = Polar Ural (0.06), 4 = Tyrol (−0.07), 3 = Lötschental (0.13) and 2 = Pyrenees (0.00). (**B**) Correlation of the reconstruction against gridded JJA SST (1901–2003).
(EPS)

Figure S4 Spatial field correlation (r>0.3) between the new MXD-based reconstruction of northern Scandinavian summer temperature and gridded (5°×5°) SLP [HadSLP2r; 20] calculated for the June–August season and 1860–2006 period.
(EPS)

Figure S5 Spatial composite analysis at 500-hPa geopotential height of (**A**) the 20 coldest and (**B**) the 20 warmest reconstructed summers (JJA; 1659–1999), and (**C**) temporal comparison (1650–1975) of the reconstructed JJA temperatures (orange) with JJA estimates of the AO (light blue). Smoothed lines are 30-year low-pass filters and stars refer to the annual extremes composed.
(EPS)

Figure S6 Temperature reconstructions from Scandinavia (green) and the Northern Hemisphere that were 60-year low-pass filtered and standardized over their common period 831–1992. The two Northern Hemisphere versions either include (brown) or exclude (ochre) Scandinavian proxies.
(EPS)

Table S1 Characteristics of the (**A**) 25 MXD chronologies and (**B**) eight instrumental stations used. Grey site codes refer to less-sensitive chronologies, blue (green) refers to updated (pre-2000) site. The mean inter-series correlation (Rbar) and the Expressed Population Signal (EPS) were computed over 30-year windows lagged by 15 years [14]. Setting refers to the number of inhabitants if a station is located in urban terrain.
(EPS)

Table S2 (**A**) Temperature extremes of the target and proxy data computed over the common period 1860–2006. Italic letters refer to common extremes. (**B**) Reconstructed summer temperature extremes and associated error estimates computed over the full period 1483–2007.
(EPS)

Table S3 Summary information of the 5 Scandinavian tree-ring chronologies used for comparison with this study. ‘Season’ refers to the originally indicated response window, whereas ‘Response’ refers to correlations computed against Scandinavian JJA mean temperatures over the 1860–1970 common period.
(EPS)
