# Supplementary material for: Causes and Consequences of Past and Projected Scandinavian Summer Temperatures, 500–2100 AD
Source: PLoS One. 2011 Sep 22;6(9):e25133. doi: 10.1371/journal.pone.0025133 (PMC3178611; doi:10.1371/journal.pone.0025133)
Supplement: Text S1 — Supporting information. (DOC) [file pone.0025133.s001.doc]

**Supporting Information (SI)**

**Calibration and Verification**

The mean station location of the eight homogenized instrumental records with continuous temperature measurements spanning >70 years during the 1881-2009 period is 68°N, 27°E, and 39 m asl, which closely matches the spatial characteristics of the tree-ring network (68°N, 23°E, and 294 m asl). For calibration, the mean and variance of the proxy data were scaled to the June-August (JJA) temperature values (1860-2006) of the gridded CRUTEM3v data averaged over the region 65-70°N and 20-30°E. This procedure is the simplest among the various calibration techniques but possibly least prone to variance underestimation [1], and thus most suitable for assessing the absolute amplitude of past temperature fluctuations. Calibration/verification exercises were computed over different split periods between 1816 and 2006. Durbin-Watson (DW) [2] statistics were computed to detect lag-1 autocorrelation in the model residuals. A DW value of 2 means there is no 1st order autocorrelation in the residuals, whereas values greater (less) than 2 indicate negative (positive) autocorrelation. Reduction of error (RE) and coefficient of efficiency (CE) statistics were applied to assess temporal stability in the calibration models. Both RE and CE statistics measure the shared variance between the actual and estimated series. CE is a more rigorous verification statistic, with all positive values demonstrating that the reconstruction has some skill [3].

**Error Estimation**

The reconstruction error was estimated by summing the errors associated with the different dataset aggregations and therefore include consideration of site location, species response, lakeshore position (distance between tree growth and the nearest lake), tree age, sampling design, growth standardization, and model calibration [4]. The range of minimum and maximum MXD indices obtained for each year between 1483 and 2006 was calculated from a pool of 25 site and 11 subset chronologies after composite RCS detrending, as well as individual Hugershoff (HUG), negative exponential (NEG) and spline (SPL) detrending. In addition to biases stemming from the various site locations included, the subset (splitting) approach accounts for possible influences of the different conifer species (pine/spruce), site ecologies (lakeshore/off-shore; i.e. trees located </> 5m from the lakeshore), juvenile, mature and adult tree ages (<100 years, 100-200 years and >200 years), as well as the sampling designs (pre/post 2000). HUG detrending utilized growth functions with positive slopes; NEG excluded such curves but computed the long-term mean instead. SPL was based on the application of cubic smoothing splines with 50% frequency-response cut-off at 300-year long waveforms. These methods are typically used for the detrending of northern tree-ring data (HUG, EXP, SPL) or to allow lower frequency information to be preserved (RCS) in resulting tree-ring chronologies [see 5 for a synthesis]. The obtained envelope of annually resolved minimum and maximum MXD indices was than combined with the two-tailed 95% bootstrap confidence band (from 1000 replacements), and added to the root mean square error (RMSE) of the calibration period (1860-2004). It should be noted that all data- and method-related errors vary over time, whereas the calibration-based RMSE describes a symmetric constant only.

**Model simulation**

The CCSM3 model [6,7], obtained from the National Centre for Atmospheric Research (http://www.ccsm.ucar.edu/), consists of atmosphere, ocean, sea-ice, and land surface components coupled without additional flux corrections [8]. The transient climate simulations of the past five centuries were externally forced by total solar irradiance (TSI), volcanic aerosol, and atmospheric greenhouse-gas (GHG) concentrations (CO_2_, N_2_O, CH_4_, and halocarbons). Solar variability as introduced by [9] was slightly rescaled towards an increase in TSI of 0.26% from the Maunder Minimum to today [6]. Volcanic aerosol was converted from TSI [9] to total aerosol masses [10], and spatially redistributed in the stratosphere [6]. GHG concentrations up to 1970 were derived from [11], whereas direct atmospheric measurements were utilized for the last 30 years of the 20th century [12]. Anthropogenic sulphate emissions, relevant to the mid-20th century, were not considered. The SRES A2 emission scenario was used for the 21st century projection [13]. The simulated SATs (65-70°N and 15-35°E) and SSTs (60-70°N and 0-10°E) were extracted from the ensemble of four transient simulations and compared with the proxy records.

**Large-scale comparison**

Linking the proxy-based northern Scandinavian temperature variations to those reported for the northern hemisphere might supply additional insight on the spatial signature of our new reconstruction. We therefore employed a variance-adjusted record of extra-tropical northern hemisphere temperatures that contains 14 individual tree-ring chronologies from Europe, Siberia and North America, as well as a newly developed ‘Scandinavian-free’ subset version of this record to avoid data overlap (initial contributions from Torneträsk, Jämtland, and Gotland were excluded). First smoothing the individual records and then normalizing them over their common period resulted in overall similar amplitudes and fairly small differences on decadal-scales (Figure S6). This methodological treatment allows comparison of regional differences in the temporal evolution of the reconstructed temperatures, but eliminates any information on possible amplitude differences between the regional- and large-scale approaches. The two large-scale reconstructions, either based on 14 or 11 site chronologies, are fairly comparable, except during the 13th century when the exclusion of generally ‘cooler’ Scandinavian proxies resulted in a warmer hemispheric mean. While the reconstructed Scandinavian temperatures were equally high during medieval times, in the early 15th century and again after ~1930, the hemispheric 20th century warming remains unprecedented in the context of the last millennium.

**Table S1.** Characteristics of the **(A)** 25 MXD chronologies and **(B)** eight instrumental stations used. Grey site codes refer to less-sensitive chronologies, blue (green) refers to updated (pre-2000) site. The mean inter-series correlation (Rbar) and the Expressed Population Signal (EPS) were computed over 30-year windows lagged by 15 years [14]. Setting refers to the number of inhabitants if a station is located in urban terrain.

**Table S2.** **(A)** Temperature extremes of the target and proxy data computed over the common period 1860-2006. Italic letters refer to common extremes. **(B)** Reconstructed summer temperature extremes and associated error estimates computed over the full period 1483-2007.

**Table S3.** Summary information of the 5 Scandinavian tree-ring chronologies used for comparison with this study. ‘Season’ refers to the originally indicated response window, whereas ‘Response’ refers to correlations computed against Scandinavian JJA mean temperatures over the 1860-1970 common period.

**Figure S1.** Temporal coverage of the 1,179 MXD series, of which 387 were developed before 2000 AD with 792 added in 2006, covers the entire Scandinavian peninsula north of 65°N and has a mean segment length (MSL) of 161 years with an maximum latewood density (MXD) of 0.72 g/cm^3^ (inset denotes the relationship between tree age and MXD value). Site chronologies start between 1400 and 1844, and their autocorrelation ranges from 0.23-0.50.

**Figure S2.** Correlation of the 25 MXD site chronologies after RCS detrending (circles) with monthly temperature means (CRUTEM3v) computed over the common 100yr interval (1860-1977). Results are classified into 20 sensitive (green) and 5 less sensitive (grey; GLO, LOF, NAR, SKI, SKS) sites, with the horizontal lines referring to their average response.

**Figure S3. (A)** Correlation (*r* >0.3) of the reconstruction against gridded JJA SAT (1901-2006) and four MXD-based summer temperature records: 1 = Polar Ural (0.06), 4 = Tyrol (-0.07), 3 = Lötschental (0.13) and 2 = Pyrenees (0.00). **(B)** Correlation of the reconstruction against gridded JJA SST (1901-2003).

**Figure S4.** Spatial field correlation (*r* >0.3) between the new MXD-based reconstruction of northern Scandinavian summer temperature and gridded (5°×5°) SLP [HadSLP2r; 20] calculated for the June-August season and 1860-2006 period.

**Figure S5.** Spatial composite analysis at 500-hPa geopotential height of **(A)** the 20 coldest and **(B)** the 20 warmest reconstructed summers (JJA; 1659-1999), and **(C)** temporal comparison (1650-1975) of the reconstructed JJA temperatures (orange) with JJA estimates of the AO (light blue). Smoothed lines are 30-year low-pass filters and stars refer to the annual extremes composed.

**Figure S6.** Temperature reconstructions from Scandinavia (green) and the Northern Hemisphere that were 60-year low-pass filtered and standardized over their common period 831-1992. The two Northern Hemisphere versions either include (brown) or exclude (ochre) Scandinavian proxies.

**SI References**

1. Esper J, Frank DC, Wilson RJS, Briffa KR (2005) Effect of scaling and regression on reconstructed temperature amplitude for the past millennium. Geophys Res Lett 32: doi10.1029/2004GL021236.

2. Durbin J, Watson GS (1951) Testing for serial correlation in least squares regression. Biometrika 38: 159-78.

3. Cook ER, Briffa KR, Jones PD (1994) Spatial regression methods in dendroclimatology: a review and comparison of two techniques. Int J Climatol 14: 379-402.

4. Esper J, Frank DC, Büntgen U, Verstege A, Luterbacher J, Xoplaki E (2007) Long-term drought severity changes in Morocco, Northwestern Africa. Geophys Res Lett 34: doi10.1029/2007GL030844.

5. Esper J, Frank D, Büntgen U, Verstege A, Hantemirov RM, Kirdyanov A (2010) Trends and uncertainties in Siberian indicators of 20th century warming. Glob Change Biol 16: 386-398.

6. Yoshimori M, Raible CC, Stocker TF, Renold M (2010) Simulated decadal oscillations of the Atlantic meridional overturning circulation in a cold climate state. Clim Dyn 34: 101-121.

7. Hofer D, Raible CC, Stocker TF (2011) Variations of the Atlantic meridional overturning circulation in control and transient simulations of the last millennium. Climate of the Past 7: 133-150.

8. Collins WD, Lee-Taylor JM, Edwards DP, Francis GL (2006) Effects of increased near-infrared absorption by water vapor on the climate system. J Geophys Res 111: doi:10.1029/2005JD006796.

9. Crowley TJ (2000) Causes of climate change over the past 1000 years. Science 289: 270-277.

10. Ammann CM, Meehl GA, Washington WM, Zender CS (2003) A monthly and latitudinally varying volcanic forcing dataset in simulations of 20th century climate. Geophys Res Lett 30: 1657.

11. Flückiger J, et al. (2002) High-resolution Holocene N2O ice core record and its relationship with CH4 and CO2. Glob Biogeochem Cycl 16: 101-108.

12. Keeling CD, Whorf TP (2005) Atmospheric CO_2_ records from sites in the SIO air sampling network. In Trends: A Compendium of Data on Global Change, Carbon Dioxide Information Analysis Center, Oak Ridge National Laboratory, U.S. Department of Energy, Oak Ridge, TN

13. IPCC 2007 Climate Change 2007: The Physical Science Basis. Contribution of Working Group I to the Fourth Assessment Report of the IPCC. Cambridge University Press, Cambridge, United Kingdom and New York.

14. Wigley TML, Briffa KR, Jones PD (1984) On the average of value of correlated time series, with applications in dendroclimatology and hydrometeorology. J Climatol Appl Meteorol 23: 201-213.

15. Briffa KR, Jones PD, Bartholin TS, Eckstein D, Schweingruber FH, Karlén W, Zetterberg P, Eronen M (1992) Fennoscandian summers from AD 500: temperature changes on short and long timescales. Clim Dyn 7: 111-119.

16. Grudd H, Briffa KR, Karlén W, Bartholin TS, Jones PD, Kromer B (2002) A 7400-year tree-ring chronology in northern Swedish Lapland: natural climatic variability expressed on annual to millennial timescales. Holocene 12: 657-665.

17. Grudd H (2008) Torneträsk tree-ring width and density AD 500-2004: a test of climatic sensitivity and a new 1500-year reconstruction of north Fennoscandian summers. Clim Dyn 31: 843-857.

18. Gouirand I, Linderholm HW, Moberg A, Wohlfarth B (2008) On the spatiotemporal characteristics of Fennoscandia tree-ring based summer temperature reconstructions. Theor Appl Climatol 91: 1-25.

18. Helama S, et al. (2009) Summer temperature variations in Lapland during the Medieval Warm Period and the Little Ice Age relative to natural instability of the thermohaline circulation on multi-decadal and multicentennial scales. J Quat Sci 24: 450-456.

20. Allan R, Ansell T (2006) A New Globally Complete Monthly Historical Gridded Mean Sea Level Pressure Dataset (HadSLP2): 1850–2004. J Clim 19: 5816-5842.
